# An Integrated Approach of Mechanistic-Modeling and Machine-Learning for Thickness Optimization of Frozen Microwaveable Foods

**DOI:** 10.3390/foods10040763

**Published:** 2021-04-03

**Authors:** Ran Yang, Zhenbo Wang, Jiajia Chen

**Affiliations:** 1 Department of Food Science, University of Tennessee, Knoxville, TN 37996, USA; ryang17@vols.utk.edu; 2 Department of Mechanical, Aerospace and Biomedical Engineering, University of Tennessee, Knoxville, TN 37996, USA; zwang124@utk.edu

**Keywords:** mechanistic-modeling, machine-learning, microwaveable food design, Bayesian optimization, thickness, heating uniformity

## Abstract

Mechanistic-modeling has been a useful tool to help food scientists in understanding complicated microwave-food interactions, but it cannot be directly used by the food developers for food design due to its resource-intensive characteristic. This study developed and validated an integrated approach that coupled mechanistic-modeling and machine-learning to achieve efficient food product design (thickness optimization) with better heating uniformity. The mechanistic-modeling that incorporated electromagnetics and heat transfer was previously developed and validated extensively and was used directly in this study. A Bayesian optimization machine-learning algorithm was developed and integrated with the mechanistic-modeling. The integrated approach was validated by comparing the optimization performance with a parametric sweep approach, which is solely based on mechanistic-modeling. The results showed that the integrated approach had the capability and robustness to optimize the thickness of different-shape products using different initial training datasets with higher efficiency (45.9% to 62.1% improvement) than the parametric sweep approach. Three rectangular-shape trays with one optimized thickness (1.56 cm) and two non-optimized thicknesses (1.20 and 2.00 cm) were 3-D printed and used in microwave heating experiments, which confirmed the feasibility of the integrated approach in thickness optimization. The integrated approach can be further developed and extended as a platform to efficiently design complicated microwavable foods with multiple-parameter optimization.

## 1. Introduction

Microwaveable food products are well accepted in the instant food market. The global microwaveable food market was evaluated at over $104 billion in 2017 and was expected to reach approximately $142 billion by 2024 [1]. However, non-uniform heating is still the biggest challenge in microwaveable foods, which may cause severe food safety and quality issues. This non-uniform heating may be improved by designing the food products with proper dimensions to improve the microwave-food interactions [2,3,4].

Mechanistic models of microwave heating have been widely used to understand the microwave-food interactions. Early-stage one-dimensional (1-D) models [2,4,5,6,7] and 2-D models [8,9,10,11] typically assumed that the microwave behaved as a planar wave and propagated into the food product in one direction. These simple models showed some insights into the complicated interactions between microwaves and food products. More recent models have been developed by incorporating the 3-D geometries of food products and oven cavity using coupled electromagnetics and heat transfer physics to evaluate the effect of food shapes and sizes [12], food placement [13], rotation of food products [14,15,16,17], material properties [13], microwave power levels [13], and microwave frequency [18,19] on the microwave heating performance. More comprehensive microwave and heat-mass transfer models that combined electromagnetics, heat transfer, moisture transport, and moisture evaporation have been developed for mashed potato [20,21] and frozen lasagna [22]. These model results significantly enhanced our understanding of the microwave-food interactions.

Although the mechanistic models with different assumptions and complexities have been good tools to understand microwave-food interactions, there are significant limitations for these models to be applied in the food industry for real food product (geometry) design. The most important limiting factor for applying these models in food design is that those modeling tools often generate a large dataset of results during the long-time simulation process, which makes it difficult for food developers to gain meaningful insights from these simulations. The mechanistic models alone often need to be evaluated at a variety of processing conditions before providing design direction to the food product developers, but not the exact product designs (e.g., dimensions). Performing mechanistic modeling becomes another type of “trial-and-error” approach to the food developers who previously prepare and heat food prototypes in ovens. Thus, there is a need to develop an enhanced approach that can better utilize the modeling results to guide the food product developers in designing the geometry of the microwavable food in a more efficient manner.

Microwaveable food geometry design can be considered as a parametric optimization problem, where the objective is to properly design the food parameters, such as shape and size, for an improved heating performance. In general parametric optimization, machine-learning has shown its capability and advantages as an efficient method to ‘self-train’ and ‘speculate’ based on limited (training) data [23,24,25]. Machine-learning models can be developed to reveal the relationship between data inputs and outputs of a system [26] and identify the proper inputs that could generate the desired outputs [27]. As discussed earlier, mechanistic modeling can generate data (e.g., heating uniformity at various geometric conditions) that could be effectively used by machine learning models to reveal the relationship and identify the optimized geometry design. Therefore, it is promising to integrate mechanistic-modeling and machine-learning to be a development tool for designing (optimizing) microwaveable food product geometry in an efficient manner.

The integrated optimization approach that combines mechanistic-modeling and machine-learning has been applied in various fields for parameter optimization, such as electronic system design [24], additive manufacture [28], solid mechanics [29], fluid flow [30], precision medicine [31], and biomedical engineering [32]. For example, by coupling machine-learning with electric-thermal simulation, the optimal conditions (e.g., airflow, material, and geometry) for 3-D integrated circuits and systems were explored by Park et al. [24]. In that study, a Bayesian optimization-based machine-learning approach was successfully developed to optimize the operation parameters and system designs, including airflow velocity, thermal conductivity of the thermal interface material, thermal conductivity of under-fill material, thermal conductivity of the printed circuit board, and thickness of thermal interface material, to minimize the maximum temperature and temperature gradient. However, a similar approach has not been reported in the food engineering area.

In this study, the thickness of microwavable food products was selected as a parameter to be optimized to achieve better heating uniformity. In geometric design, the thickness of the microwavable food product is the most critical parameter that influences the microwave heating uniformity. Chamchong & Datta [33] evaluated the effect of load geometry on the microwave thawing performance of frozen products and found that the product with a lower aspect ratio (thinner thickness) could be thawed in a shorter time with better temperature uniformity. Bhattacharya & Basak [34] performed a comprehensive analysis of the effect of shape on the microwave heating dynamics of food materials. The results showed that the thickness of the sample influences the average power absorption and temperature distribution significantly for different shapes and food materials. Therefore, it is a good start to develop an integrated mechanistic-modeling and machine-learning framework to optimize the thickness of microwaveable food products for improved heating uniformity.

The objectives of this study are to:

(1) develop and validate an integrated framework that combines mechanistic-modeling and machine-learning to optimize product thickness with best heating uniformity;
(2) evaluate the robustness of the integrated mechanistic-modeling and machine-learning approach for different scenarios of initial training datasets and food product shapes; and
(3) compare the optimization results and efficiency between the integrated and parametric sweep (modeling alone) approaches.

## 2. Materials and Methods

### 2.1. Mechanistic-Modeling

**Figure 1:**
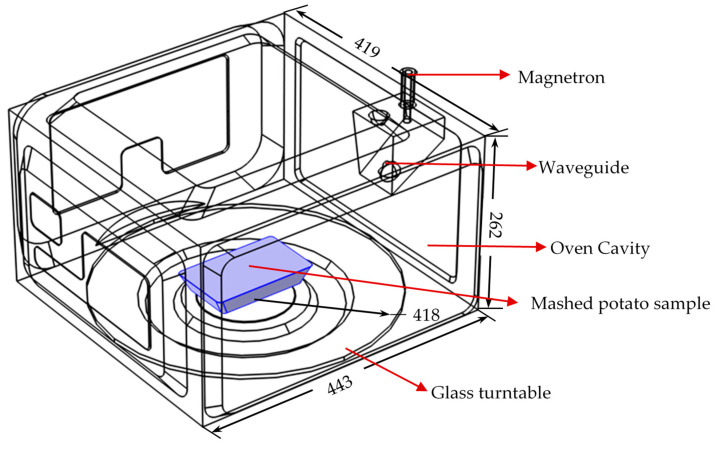
Geometric model of microwave oven and mashed potato in the mechanistic-modeling. (Unit: mm).

**Figure 2:**
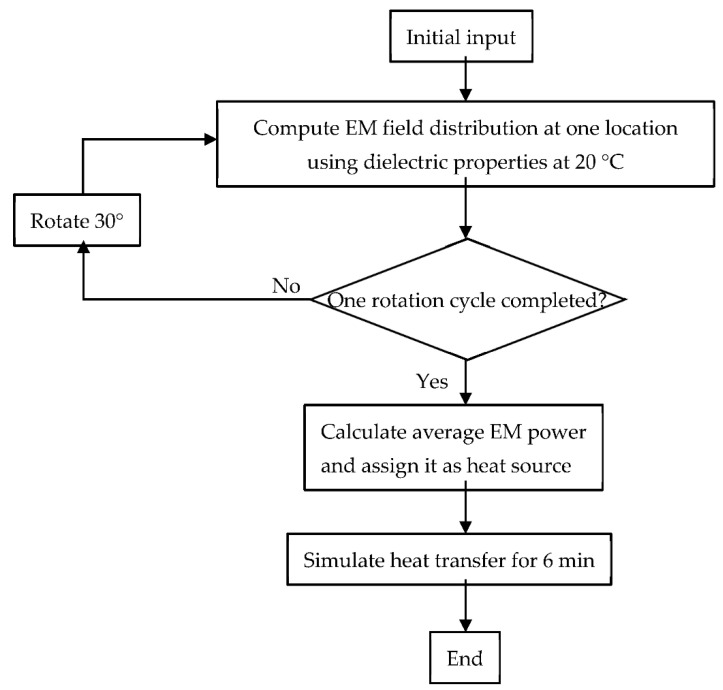
Flow chart of the microwave heating simulation based on the strategy with discrete rotation and decoupled approach of electromagnetic (EM) field and heat transfer.

Many mechanistic models for the microwave heating process have been extensively developed and validated. This study used a previously developed and validated mechanistic model to simulate the microwave heating process. To avoid the repeatability of model development and help the reader focus on the integrated approach developed in this study, the mechanistic model geometry, governing equations, boundary conditions, and simulation strategies were not discussed in detail here but only summarized as follows. The model was developed in a finite-element-method-based commercial software, COMSOL Multiphysics v5.5. Generally, the domestic microwave oven (Model no: NN-SD9675, Panasonic Corporation, Shanghai, China) was used, as shown in Figure 1. The physics include electromagnetics of Maxwell’s equations and Fourier’s heat transfer equation. The rotation of the food product (mashed potato) was included using a discrete moving step approach [21], where 12 discrete locations were used to represent one rotational cycle. In order to reduce the total computational time, a decoupled approach [35] was used to simulate the electromagnetic (EM) field and the temperature distribution. This decoupled approach was comprehensively evaluated and found that the use of room-temperature dielectric properties throughout the whole modeling process did not influence the model prediction accuracy much but could significantly reduce the computational time, for some types of food products with relatively stable dielectric properties from thawed to cooked temperatures. Typically, high moisture and low salt contents foods meet the requirement, including the mashed potato used in this study. The simulation approach was shown in Figure 2. The model parameters and material properties were summarized in Table 1.

The mechanistic-modeling simulation was performed on a Microway workstation (Microway, Inc., Plymouth, MA, USA) with a 128 GB RAM operating memory running on two 16-core Intel(R) Xeon(R) 3.20 GHz. The computational time for simulating one 6-min microwave heating process using the decoupled approach was about 1 h.

### 2.2. Parametric Sweep of Mechanistic-Modeling Approach to Determine Optimal Thickness with “Best” Heating Uniformity

A parametric sweep approach based on mechanistic modeling was used to determine the optimal design (thickness) of a food product with “best” heating uniformity.

In this study, the thickness of a rectangular frustum-shaped food product (mashed potato) with a constant volume of 300 mL was to be optimized. This sample size was selected because a typical microwaveable meal on the market is 9 oz (266 mL) to 11 oz (325 mL).

The volume of the rectangular frustum-shaped product can be described by Equation (1):

(1) $$V=\frac{d}{3}\left(L_{Top}W_{Top}+L_{Bottom}W_{Bottom}+\sqrt{L_{Top}W_{Top}\times L_{Bottom}W_{Bottom}}\right)$$

where *V* is the volume of the frustum, *d* is the thickness of the frustum, *L_Top_* and *W_Top_* are the length and width of the top surface, *L_Bottom_* and *W_Bottom_* are the length and width of the bottom surface, respectively.

The specific side length relationships were defined in Equations (2)–(4):

(2) $$L_{Top}=1.2L_{Bottom}$$

(3) $$W_{Top}=1.2W_{Bottom}$$

(4) $$W_{Bottom}=0.6L_{Bottom}$$

We have

(5) $$V=\;0.728\times d\times L_{Bottom}^{2}$$

(6) $$L_{Bottom}=\sqrt{\frac{V}{0.728d}}$$

When the thickness of the food (*d*) is defined, the *L_Bottom_* and the exact size of the food can be determined.

The heating uniformity of the food product design was the objective function of the optimization problem. The heating uniformity index (HUI) of a food product at a given thickness can be described by the ratio of standard deviation and average temperature rise inside the food product [37] using Equation (7):

(7) $$\mathrm{HUI}=\;\frac{T_{std}}{T_{ave}-T_{initial}}$$

where the *T_ave_* is the final volumetric average temperature (°C), *T_initial_* is the initial temperature (−10 °C), *T_std_* is the standard deviation of temperature during the heating process, defined as:

(8) $$T_{std}=\;\frac{1}{V_{vol}}\int_{V_{vol}}\sqrt{{\left(T_{local}-T_{ave}\right)}^{2}}dV_{vol}$$

where *V_vol_* is the sample volume, *T_local_* is the local temperature.

The goal of this optimization process was to find an optimal thickness with the best heating uniformity (lowest HUI). In order to achieve this goal, a parametric sweep approach of mechanistic-modeling was used to manually setup thickness values and evaluate the heating uniformity at discrete thicknesses of the food product. In this study, the thickness was swept from 1.2 cm to 4.0 cm at a step size of 0.1 cm, and a total of 29 thicknesses were evaluated. This thickness sweep range was selected because it represented a reasonable microwaveable food product thickness (~0.5 to ~1.5 inches) that may have better heating uniformity than other thicknesses. The determined optimal thickness from the parametric sweep approach was used as a reference to validate the integrated mechanistic-modeling and machine-learning approach that will be described below.

### 2.3. Integrated Mechanistic-Modeling and Machine-Learning Approach to Optimize Thickness

**Figure 3:**
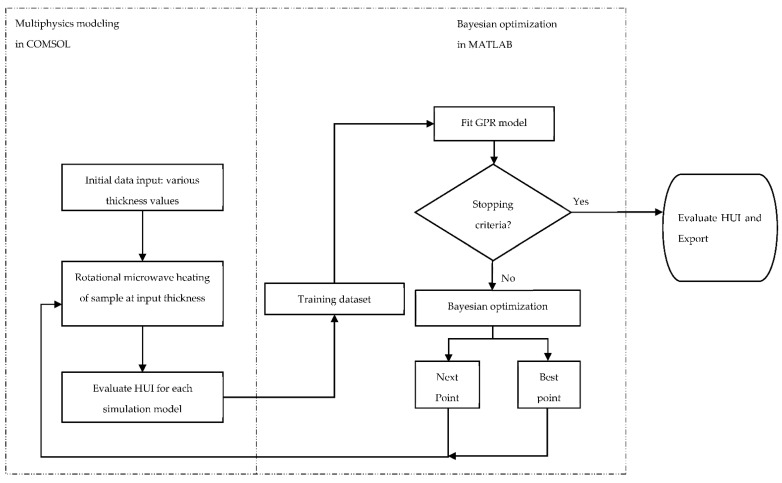
Proposed integrated approach that combines mechanistic-modeling and Bayesian optimization-based machine-learning. (GPR: Gaussian process regression; HUI: Heating uniformity index).

In order to find a thickness with the best heating uniformity (lowest HUI), an integrated approach that coupled mechanistic-modeling and machine-learning was developed (Figure 3) and validated by comparing the optimization result to that of the parametric sweep approach. The mechanistic-modeling section was developed following the procedures described in Section 2.1. The major function of the mechanistic-modeling was to simulate the microwave heating performance at a given thickness and provide datasets of thickness and HUI results as training data to the machine-learning section. The machine-learning section was implemented in MATLAB 2020a (The MathWorks, Inc., Natick, MA, USA) and its major function was to predict the “best thickness” with the lowest HUI and provide learned and updated data points to guide the mechanistic-modeling section. The mechanistic-modeling and machine-learning were performed in cycles until an optimized thickness was determined. The integration of mechanistic-modeling and machine-learning was achieved through the COMSOL-MATLAB livelink^TM^.

#### 2.3.1. Bayesian Optimization-Based Machine-Learning Strategy

The optimization in the machine-learning section was performed using a Bayesian optimization strategy, including three components: a training dataset, a nonparametric model fitted with training data, and learned-for-update data points. In one optimization step, the optimization algorithm could use the training dataset to fit a nonparametric model and identify the “best point” and “next point” that could be used to improve the representativeness of the training dataset.

In this study, a group of thickness~HUI data obtained from the mechanistic-modeling was used as the initial training input, referred to as the initial dataset P_0_. The initial thickness values were evenly selected between 1.2 and 4.0 cm. The corresponding HUI values at the selected thicknesses were determined by the mechanistic models. The paired thickness and HUI values were imported into the Bayesian optimization algorithm to train the initial model. Five, seven, and nine pairs of thickness~HUI initial datasets were evaluated to determine the optimal initial dataset size based on the optimization performance (accuracy and efficiency).

In the Bayesian optimization, the training dataset was fitted to a nonparametric Gaussian Process Regression (GPR) model as a function of input parameters [25]. The GPR model is a probabilistic model that cannot be expressed as any specific functions. It is built based on the assumption that all input parameters are from a group of random variables, such that any finite number of them follow Gaussian distribution. The random variable set forms a Gaussian process (GP). If $\left\{f\left(x\right),\;x\in {\mathbb{R}}^{d}\right\}$ is a GP, then given n samples $\left\{x_{1},x_{2},\ldots ,x_{n}\right\}$, the distribution of $\left\{f\left(x_{1}\right),\;f\left(x_{2}\right),\;\ldots ,f\left(x_{n}\right)\right\}$ is Gaussian [38]. The strategy applied to the model fitting process is to randomly grab observation samples within the defined range (1.2 to 4 cm in this study) that follows Gaussian distribution and add these thicknesses to the GPR model. The observation samples were selected by the built-in acquisition function during the fitting process. The most commonly used acquisition functions are expected improvement (EI), probability of improvement (PI), and upper/lower confidence bound (UCB/LCB) [24]. This function randomly selected thickness value, following a defined exploration-exploitation ratio (ER), within the thickness range and predicted the corresponding HUI values following the GP rule. With more iterations in one optimization step to find such thickness, the prediction model is more specific. The default setting of 30 iterations in MATLAB Bayesian Optimization function and ER value of 0.5 were used in this work. After one optimization step, the estimated “best thickness” with the estimated “lowest HUI” based on the current training dataset and the “next thickness” with high potential to increase the confidence of the training model were determined.

#### 2.3.2. Integrating Bayesian Optimization-Based Machine-Learning with Mechanistic-Modeling for Thickness Optimization

After one Bayesian optimization step with the estimated “best thickness” and “next thickness” determined, the two thickness points were evaluated in the mechanistic-modeling to determine their corresponding HUI values. Note that the estimated “best thickness” with an estimated “lowest HUI” was determined as an evaluated HUI by the mechanistic-modeling. The two pairs of evaluated thickness~ HUI were added into the previous training dataset for the next optimization step. The mechanistic-modeling and machine-learning were repeated in cycles until the following stopping criteria were met.

**Figure 4:**
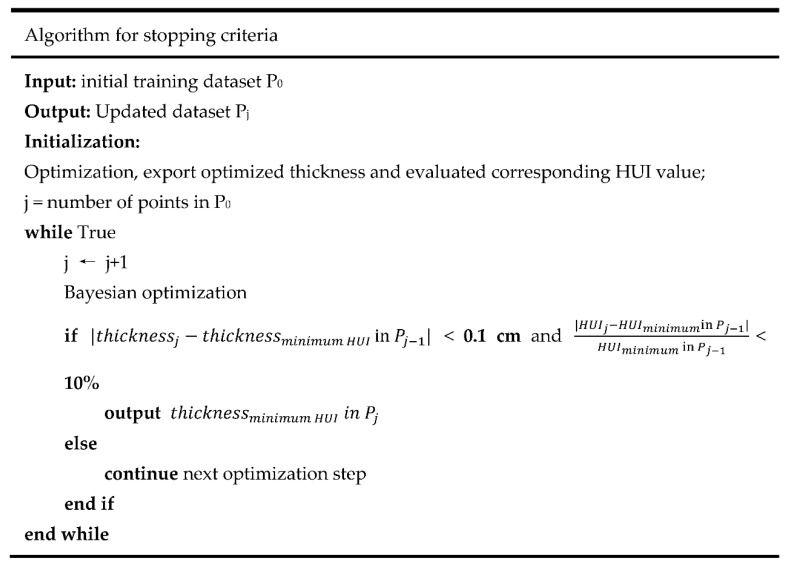
Pseudocode depicting the algorithm for stopping the integrated mechanistic-modeling and machine-learning process.

There were two criteria to satisfy before stopping the optimization process, as shown in Figure 4. (1) There was a slight difference between the last evaluated best thickness and the currently estimated “best thickness” (i.e., <0.1 cm). (2) The improvement in terms of the HUI was small enough (<10% of the last evaluated HUI value). The first criterion was set based on the real-life production situation that it is unnecessary/infeasible to control the thickness of a sample to be accurate in the millimeter range. The second criterion prevented the Bayesian optimization from over-evaluating the potential thickness with little improvement in heating uniformity. These stopping criteria could achieve a balance between the potential thickness improvement and the consumption of computational resources. The tolerance values in the stopping criteria can be modified based on the real processing precision in the food industry.

After the stopping criteria were satisfied, the mechanistic-modeling and machine-learning cycle ended. Within all simulated results, the optimized thickness was determined as the one with the lowest evaluated HUI.

### 2.4. Evaluation of the Accuracy, Robustness, and Efficiency of the Integrated Mechanistic-Modeling and Machine-Learning Approach

#### 2.4.1. Validation of the Integrated Approach

The performance of the integrated mechanistic-modeling and machine-learning approach was validated by comparing the optimized thickness and the corresponding HUI value with the values from the parametric sweep approach of mechanistic-modeling. Since the acquisition process of the machine-learning algorithm is random, each optimization process may start at different thicknesses and show slightly different optimized results. Thus, the optimization process was performed three times and validated by the parametric sweep approach.

#### 2.4.2. Evaluation of the Robustness of the Integrated Approach for Different Initial Training Datasets

In the baseline integrated approach, seven initial training data points were evenly selected between the thickness range (i.e., from 1.2 to 4 cm). To test the effect of training datasets on the optimization results (optimized thicknesses and their corresponding HUI values), a random training dataset generating strategy was also evaluated in the optimization process. All the optimization processes using the newly developed training datasets were performed three times. The HUI values of the optimized thicknesses determined from the integrated approach with different training dataset were compared with the HUI of the best thickness determined from the parametric sweep approach of mechanistic-modeling by evaluating the HUI difference using Equation (9):

(9) $$\mathrm{HUI}\;change=\frac{\left({\mathrm{HUI}}_{integrated\;approach}-{\mathrm{HUI}}_{parametric\;sweep}\right)}{{\mathrm{HUI}}_{parametric\;sweep}}\times 100\%$$

The small HUI change indicates that the integrated approach is robust to identify optimized thickness with good heating uniformity that is close to the parametric sweep approach. The negative HUI change indicates that the integrated approach could have better optimization result for more uniform heating than the parametric sweep approach.

#### 2.4.3. Evaluation of the Robustness of the Integrated Approach for Different Food Shapes

**Figure 5:**
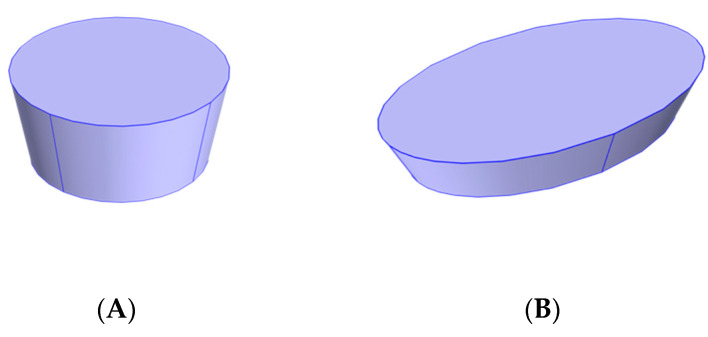
Geometry for different shaped samples for thickness optimization (**A**) conical frustum-shaped sample; (**B**) elliptical frustum-shaped sample.

In addition to the rectangular frustum-shaped product used in all above conditions, the robustness of the integrated approach was also evaluated for the conical frustum-shaped and elliptical frustum-shaped food products (as shown in Figure 5) by comparing their optimized thicknesses with the optimal ones determined by the parametric sweep approach of mechanistic-modeling. The volume of the products with different shapes was also set as constant (300 mL), while the thickness was optimized with changing side dimensions. The thickness changed between 1.2 and 4.0 cm.

For the conical frustum, the top radius was 1.2 times of the bottom radius. The relationship between the bottom radius and the thickness can be determined by Equation (10):

(10) $$V=\frac{\pi d}{3}\left(r_{Bottom}{}^{2}+r_{Top}r_{Bottom}+r_{Top}{}^{2}\right)$$

Thus, the bottom side length can be determined as:

(11) $$r_{Bottom}=\sqrt{\frac{3V}{3.64\pi d}}$$

where $r_{Bottom}$ is the radius of the bottom surface, $r_{Top}$ is the radius of the top surface, and d is the thickness of the food product.

For the elliptical frustum-shaped samples, the major and minor radii for the top surface were set as 1.2 times of the ones for the bottom surface, respectively; the minor radius was set as 60% of the major radius; and the volume was 300 mL. The relationships among volume, top surface major and minor radii, and thickness can be described by Equations (12)–(14):

(12) $$V=\frac{d}{3}\left(A_{top}+A_{bottom}+\sqrt{A_{top}A_{bottom}}\right)$$

(13) $$A_{bottom}=r_{major,b}r_{minor,b}\pi =0.6\pi r_{major,b}^{2}$$

(14) $$A_{top}=r_{major,t}r_{minor,t}\pi =0.864\pi r_{major,b}^{2}$$

where $A_{top}\;\mathrm{and}\;A_{bottom}$ are the areas of the top and bottom surfaces, respectively, *r_major,b_* and *r_minor,b_* are the major and minor radii of the bottom surface, *r_major,t_* and *r_minor,t_* are the major and minor radii of the top surface.

For these groups of samples, initial datasets with seven evenly distributed points were used in the integrated approach to optimize the thickness to achieve the best heating uniformity (lowest HUI values). The optimization process was performed three times for each shape of the food product and validated by the parametric sweep approach.

#### 2.4.4. Evaluation of the Efficiency of the Integrated Approach

The efficiency of the integrated approach was evaluated by comparing the number of mechanistic models run to achieve the optimized thickness with that of the parametric sweep approach. The efficiency improvement of the integrated approach was evaluated by Equation (15):

(15) $$Efficiency\;improvement=\frac{1-N_{integrated\;approach}}{N_{parametric\;sweep}}\times 100\%$$

where *N_integrated__approach_* is the number of mechanistic models (including initial training data models run in the integrated approach, *N_parametric sweep_* is the number of parametric sweep models (N = 29).

### 2.5. Micorwave Experimental Confirmation

**Figure 6:**
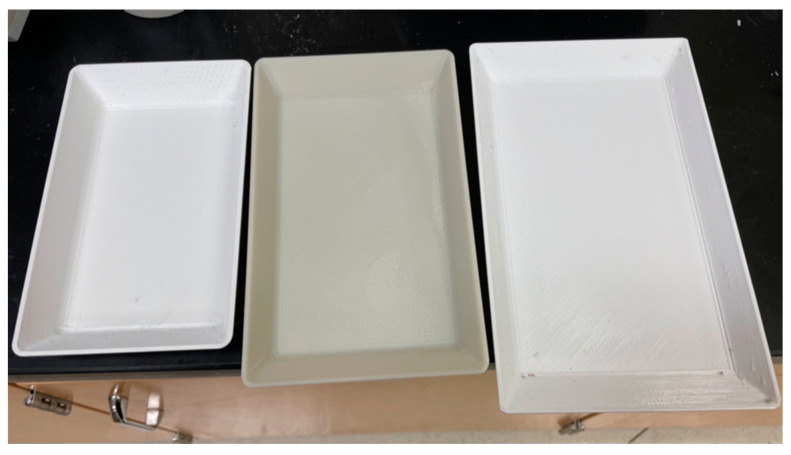
3D-printed rectangular trays with one optimized thickness (middle, 1.56 cm) and two non-optimized thicknesses (left 2.00 cm and right 1.20 cm).

In order to confirm the feasibility of the integrated approach, microwave heating experiments were performed for rectangular trays with one optimized thickness (1.56 cm) and two non-optimized thicknesses (1.20 and 2.00 cm) around the optimized thickness. The three trays with specific dimensions were made by the 3D printing technology using a uPrint SE Plus printer (Stratasys Ltd., Eden Prairie, MN, USA), as shown in Figure 6. Mashed potato samples [36] were prepared and filled in the trays for freezing at −10 °C. A slice of mesh cloth was placed at the center layer of the mashed potato samples to separate the samples to be two layers. The frozen mashed potato samples were heated in a microwave oven (Model no: NN-SD9675, Panasonic Corporation, Shanghai, China) for 6 min with full power of 1200 W. After heating, the spatial temperature profiles at the top, middle, and bottom layers were recorded by a thermal imaging camera (FLIR C3, Boston, MA, USA). The HUI values based on the combined three layers of temperature profiles were calculated and compared among three samples with different thicknesses. The microwave heating experiments were performed in duplicates.

## 3. Results and Discussion

### 3.1. Optimal Thickness Determined from the Parametric Sweep of Mechanistic-Modeling

**Figure 7:**
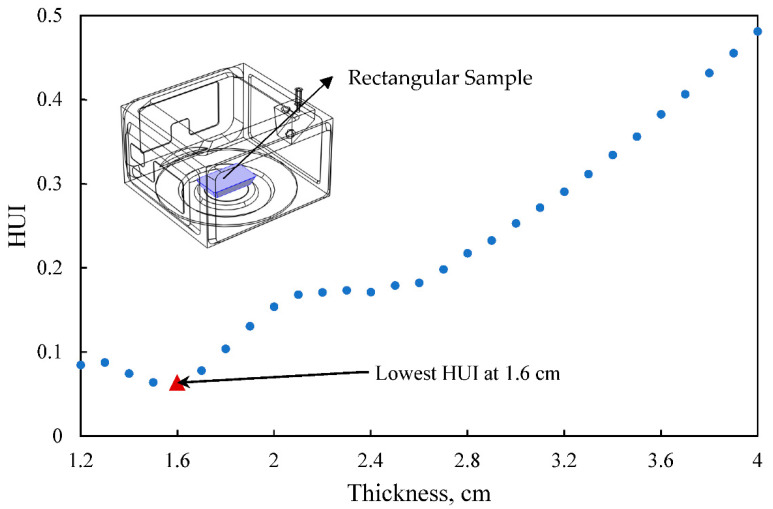
The heating uniformity index (HUI) of temperature at different thicknesses determined by using the parametric sweep approach of mechanistic-modeling for thickness sweeping from 1.2 to 4.0 cm with a step size of 0.1 cm.

The thickness of the rectangular frustum-shaped product was swept from 1.2 to 4.0 cm with a step size of 0.1 cm using mechanistic-modeling to determine the corresponding HUI values. The 29 thickness values and their corresponding HUI values were plotted in Figure 7. Generally, the HUI values decreased with thickness at thin thicknesses range and then increased with thickness increasing. The lowest HUI value was reported as ~0.064 at the thickness of 1.60 cm, indicating that the approximate optimal thickness with the best heating uniformity can be determined around this thickness. In microwaveable food development, the penetration depth is an important parameter to estimate the suitable thickness of the food product for a relatively uniform heating result [39]. It is often recommended that the thickness of a microwavable food product should be less than 2~3 times the penetration depth for minimizing surface heating [40]. In this study, the penetration depth of mashed potato decreased significantly from about 3.3 cm at −10 °C to about 5 mm after thawing at 2.45 GHz [35]. The optimal thickness of 1.60 cm determined by the parametric sweep approach was within a reasonable range for better heating uniformity. In this study, the optimal thickness determined from the parametric sweep approach was used as a “true value” to validate the integrated approach. Note that in the parametric sweep plot, the function of HUI was not increasing monotonically. Within the range of 1.3 to 1.6 cm and 2.3 to 2.4 cm, the HUI value decreased as the thickness increased. Therefore, a finer sweep step is necessary for the parametric sweep approach to find the global minimum value.

### 3.2. Optimized Thickness Determined from the Integrated Approach

To fit the probabilistic GPR model, there is no specific number of training data points for the training dataset; hence we arbitrarily use five, seven, and nine evenly distributed data points to perform the optimization. All of these three optimization processes were repeated three times separately for a generalized performance assessment.

#### 3.2.1. Optimal Number of Initial Training Points

**Figure 8:**
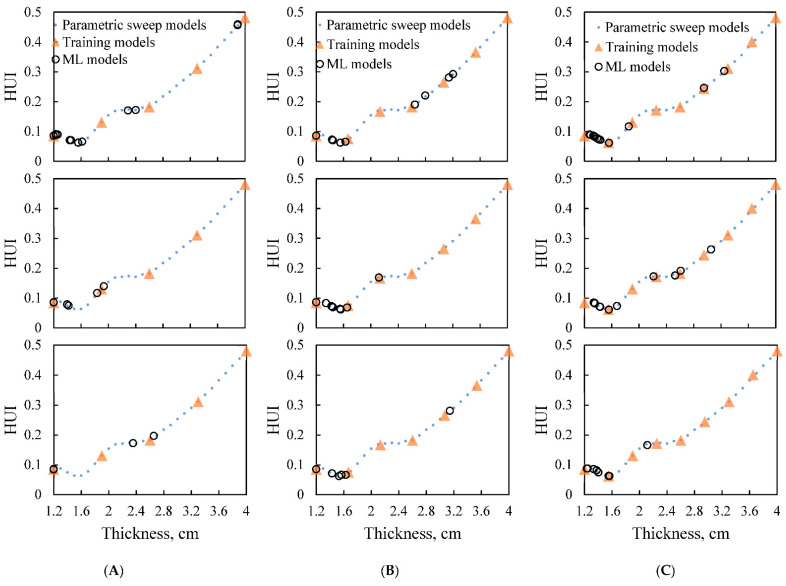
Machine-learning (ML) optimization process with evenly distributed initial training dataset consists of (**A**) five, (**B**) seven and (**C**) nine points for the rectangular frustum.

Various numbers of paired thicknesses and HUI values were used as the initial training dataset (Δ-shape points in Figure 8) to train the optimization model. After the initial model fitting step, the Bayesian optimization algorithm started to optimize the thickness by evaluating the HUI values at different thicknesses using mechanistic-modeling and updated these newly evaluated pairs (o-shape points in Figure 8) into the training dataset. The fitting-updating loop ended when stopping criteria were satisfied. With different numbers of initial training data points, the optimization process performance differed. When with five initial points (Figure 8A), only one of the replications succeeded in finding the thickness that is close to the lowest range. When there were seven points in the initial training step (Figure 8B), all the optimized thickness values from three replications were close to the optimal thickness determined from the parametric sweep approach. Similar optimization results were observed when nine points were used in the initial training dataset (Figure 8C). Therefore, the optimal number of initial training data points was determined as seven to be used in further evaluations.

#### 3.2.2. Optimization Results from the Integrated Approach Initialized with the Optimal Number of Training Data Points (7)

As shown in Figure 8B, the optimized thickness can all be determined to be around 1.60 cm after 6~10 steps for all three replications. The optimized thickness values determined by the integrated approach were 1.55 cm, 1.56 cm, and 1.53 cm, with HUI values of 0.062, 0.061, and 0.062, respectively, for three replications. The optimized thickness was close to the optimal one determined from the parametric sweep approach, and the difference was smaller than 0.1 cm, suggesting that the integrated approach can predict approximate and consistent “best thickness” with low heat non-uniformity that was similar to the one determined from the parametric sweep approach (thickness of 1.60 cm with HUI of 0.064). In all three replications, the optimized results were all global minimum results according to the parametric sweep plot, indicating the algorithm is a promising strategy in optimizing parameters without providing misleading answers. The optimized thicknesses from the integrated approach all had even lower HUI values (better heating uniformity) compared with the one from the parametric sweep approach, showing better results.

### 3.3. Robustness of the Integrated Approach in Optimizing the Thickness

In order to test the robustness of the integrated mechanistic-modeling and machine-learning approach, further assessments on the effect of training datasets and food product shapes on optimization results were performed.

#### 3.3.1. Effect of Training Datasets

**Figure 9:**
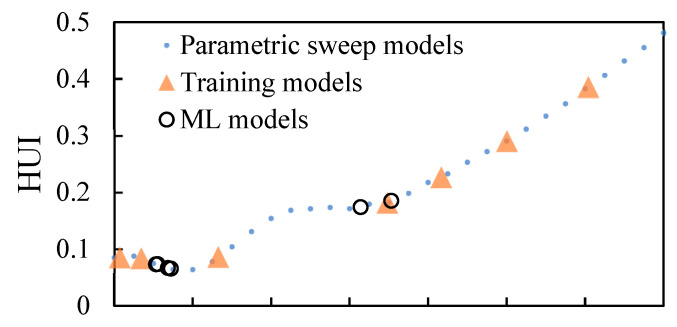

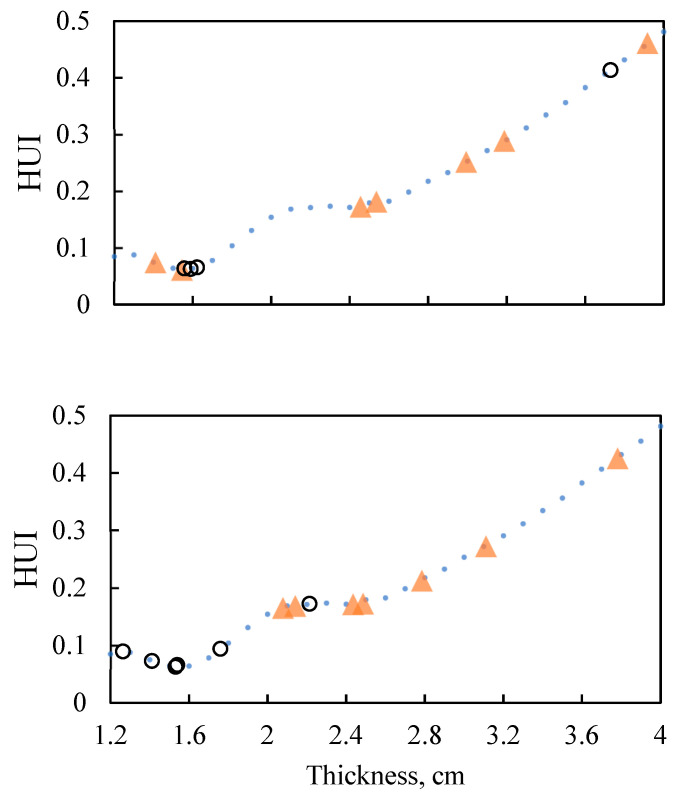
Triplicates of machine-learning optimization process with randomly selected training datasets for the rectangular frustum-shaped sample.

In the evenly distributed training datasets, the second one of the seven initial training data points (1.67 cm) was close to the optimal thickness determined from the parametric sweep approach, which might direct the machine-learning algorithm to exploit the nearby area more. To test the robustness of the integrated approach when without the (positive) influence caused by the second training point, randomly selected thickness values within the thickness range (1.2 to 4 cm) were used in the initial dataset, as shown in Figure 8. The seven thickness points were generated by the MATLAB random number function. As shown in Figure 9, the randomly selected training thickness values distributed dispersedly over the thickness range. From two out of the three replications of optimization (Figure 9), most of the evaluated data points were in the optimal range. The dispersed evaluated points in Figure 8B could be attributed to the wide blank range of unevaluated thicknesses (1.2 to ~2.1 cm) in the training dataset, which led to the focused exploration in that region. This showed the importance of a proper training dataset in the optimization process. Overall, the integrated approach using randomly selected training datasets still determined the optimized result with 4 to 6 optimization steps for three replications, showing the robustness of the integrated approach.

#### 3.3.2. Effect of Food Product Shapes

**Figure 10:**
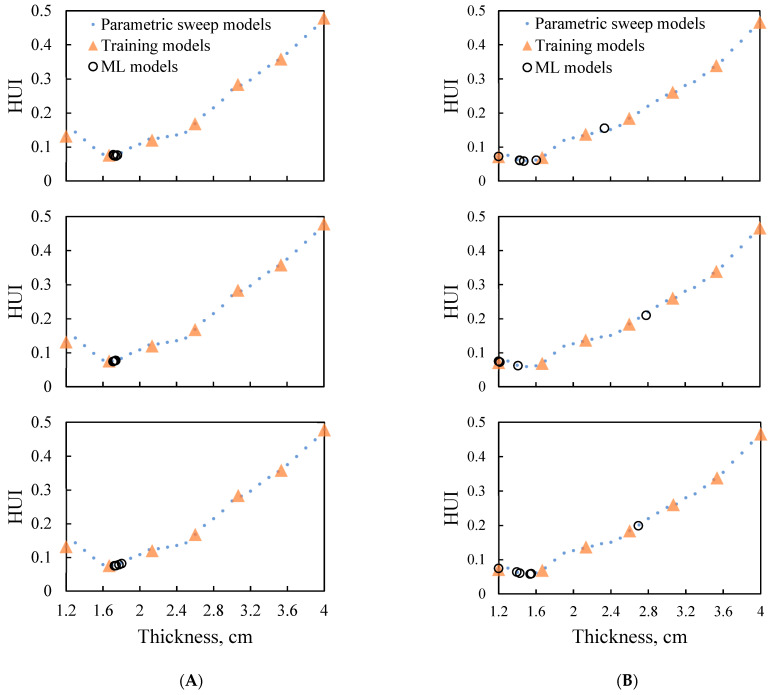
Machine-learning (ML) process with evenly distributed training datasets for (**A**) Conical frustum-shaped sample; (**B**) Elliptical frustum-shaped sample.

To further evaluate the robustness of the integrated approach, mashed potato samples in conical frustum and elliptical frustum shapes were evaluated by following the same test flow as that for the rectangular frustum sample. Only the evenly distributed initial training datasets with seven points were used. As shown in Figure 10, the HUI~thickness data determined from the parametric sweep approach for conical frustum-shaped and elliptical frustum-shaped samples were slightly different from the rectangular frustum one. While, the integrated approach was still able to determine the optimized thickness with low HUI values. The optimized thicknesses of three replications were obtained in 4 optimization steps for the conical frustum-shaped (Figure 10A) and 4 to 6 optimization steps for elliptical frustum-shaped (Figure 10B) samples. The optimized thicknesses were 1.71~1.74 cm for the conical frustum sample, whose optimal thickness determined by the parametric sweep approach was 1.70 cm. For the elliptical frustum-shaped sample, the optimized thickness ranges from 1.41 to 1.53 cm, which was close to the thickness of 1.50 cm determined by the parametric sweep approach. Thus, the integrated approach of mechanistic-modeling and machine-learning was robust for optimizing the thickness of food products with different shapes.

### 3.4. Summary of the Comparison between Integrated and Parametric Sweep Approaches

The comparison of optimization results (thickness and HUI values) and optimization efficiency between the integrated and parametric sweep approaches for all evaluated scenarios were summarized in this section.

#### 3.4.1. Optimized Thickness and Corresponding Heating Uniformity Index (HUI)

Table 2 summarized the optimized thickness using the integrated approach and corresponding HUI change compared with the parametric sweep approach. For the rectangular frustum-shaped sample optimized with the evenly distributed training dataset, the optimized thickness was between 1.53 and 1.56 cm, which is close to the optimal one (1.60 cm) determined from the parametric sweep approach. The HUI change was between −3.2% and −2.5%, where the negative HUI change indicated the heating uniformity improvement and therefore better optimized results. Thus, the parametric sweep approach could identify the lowest HUI values at evaluated discrete thickness values, while the integrated approach could potentially optimize the thickness with even better heating uniformity.

For the rectangular frustum-shaped sample optimization with a randomly selected training dataset, the optimized thickness values were also similar to the optimal one determined from the parametric sweep approach, with close HUI values (between −3.5% and +2.7%). The better performance of the randomly selected initial training dataset approach (−3.5% in Figure 9) may be attributed to that there was one training data point very close to the lowest HUI region.

The integrated approach was able to identify the optimized thickness values that were close to the optimal thickness values determined from the parametric sweep approach for the conical and elliptical frustum-shaped samples. The HUI changes were in the range of (−3.7%, −0.8%) and (−2.6%, +4.3%) for the conical and elliptical frustum-shaped samples, respectively.

In summary, the integrated approach also can optimize the thickness to have similar or better heating uniformity when compared to the parametric sweep approach. Among all scenarios, there was at least one optimization process that could find a thickness that had better uniformity (HUI) than the parametric sweep results.

#### 3.4.2. Optimization Efficiency

Table 3 summarized the average number of mechanistic models that need to be performed in the integrated optimization process of three replications. The average numbers of mechanistic models (including training models and updating models) in the integrated optimization process were between 11.0 and 15.7 for optimizing food products with different shapes using different initial training datasets. When compared to the parametric sweep approach (29 models were used), the optimization efficiency can be improved by 45.9% to 62.1%. The efficiency improvement of the integrated approach is critical for the real food product development [41], where the reduced product development cycle could save the industry’s time and resource investment and significantly enhance its competitiveness.

### 3.5. Experimental Heating Uniformity Index

**Figure 11:**
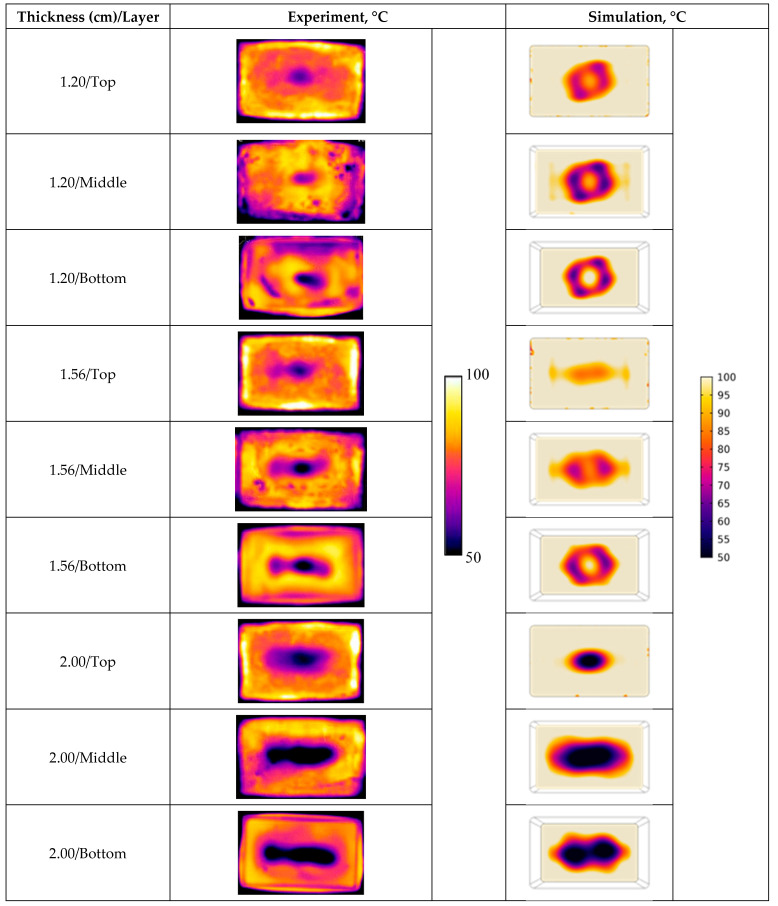
Comparison between experimental and simulated temperature profiles after six-minute heating.

The rectangular-shape trays with one optimized thickness (1.56 cm) and two non-optimized thicknesses (1.20 and 2.00 cm) were used in the microwave heating experiments. Figure 11 showed the comparison between experimental and simulated spatial temperature profiles for three thicknesses at top, middle, and bottom layers. Generally, the simulated temperature profiles matched well with the experimental results, but with slightly higher predictions. The higher temperature prediction was mainly attributed to the ignorance of moisture evaporation in the mechanistic models. The recorded experimental temperature values were also (~2 to 3 °C) lower than the real final temperature values due to the time needed to take thermal images and the moisture evaporation.

**Figure 12:**
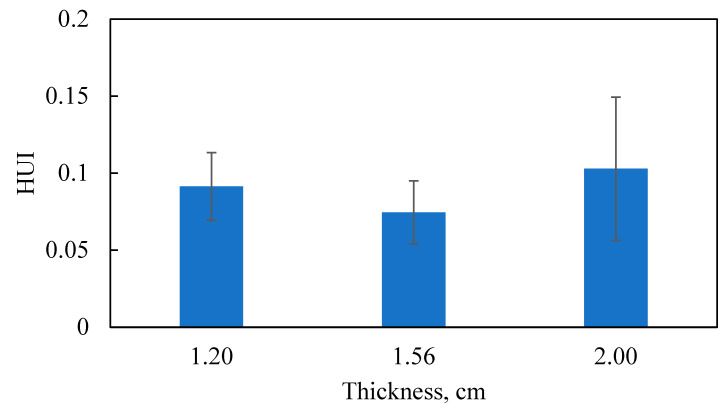
Experimental results of heating uniformity index expressed as mean ± standard deviation from duplicate.

As shown in Figure 12, the HUI value of the optimized thickness sample was smaller than those of the non-optimized thickness samples, which confirmed that the integrated approach was able to optimize the thickness with reduced HUI and improved heating uniformity. It is also noted that, the exact HUI values from experiments were slightly different from the simulation results, because the experiments were only able to obtain three layers of the samples while the simulated HUI values were evaluated for the whole tray of samples.

### 3.6. Limitations and Future Work

This study demonstrated a feasible framework that used an integrated mechanistic-modeling and machine-learning approach to optimize the thickness of frozen homogenous microwaveable foods with improved optimization results and efficiency when compared to the approach using modeling alone. There are several limitations in this study that can be addressed in future work to further develop the integrated approach.

First, the mechanistic-modeling section used a decoupled approach to simulate the microwave heating process, where room-temperature dielectric properties of mashed potato were used to simulate the electric field and microwave power absorption for saving computation time. This decoupled approach is valid for food products with little change of penetration depth from thawed to cooked temperatures. For other food products with significant change of penetration depth with temperature, a coupled approach with temperature-dependent dielectric properties might be necessary. The integrated approach of mechanistic-modeling and machine-learning can still be developed and used. Note that the updating dielectric properties during microwave heating modeling is significantly time-consuming and the integrated approach will be much more efficient than the parametric sweep approach.

Second, although this study was focused on a single parameter (thickness) optimization in one microwave oven, the optimization results demonstrated the capability, robustness, and efficiency of the integrated approach in food product development. With the foundational framework developed in this study, more complicated food design problems (e.g., multiple parameters optimization in heterogeneous foods with multiple objective functions) could be considered to further improve the proposed approach. It is our expectation that the integrated approach is more powerful when applied to the development of more complicated food products since multiple parameters may need to be optimized at the same time. In addition to the heating uniformity index, microwave power absorption and heating efficiency also can be incorporated in the objective function. Dealing with such a more complicated problem would further highlight the strength of this proposed approach, which could significantly reduce the computational load for modeling all possible product parameter combinations. This will lead to our long-term goal of developing an integrated mechanistic-modeling and machine-learning-based product development platform to enable efficient microwaveable food product design.

## 4. Conclusions

By combining mechanistic-modeling and machine-learning techniques, an integrated approach was developed and applied to thickness optimization of a homogenous frozen microwavable food with improved heating uniformity. The optimized thickness values generated by the integrated process were close to or better than the optimal thickness determined by the parametric sweep approach of mechanistic-modeling. The integrated approach can significantly reduce the number of mechanistic models by 45.9% to 62.1% when compared to the parametric sweep approach. The integrated approach was robust to deal with different initial training datasets and optimize food products with different shapes. Microwave heating experiments using 3-D printed rectangular trays confirmed the optimized result. The presented results demonstrated the capability, robustness, and efficiency of the integrated mechanistic-modeling and machine-learning approach, which was promising to be used for the development and design optimization of more complicated food products.

## Figures and Tables

**Table 1:** Parameters and conditions in mechanistic-modeling of microwave heating.

Parameter/Condition		Value/Source
Mashed potato sample	Thickness (cm)	1.2~4.0
	Initial temperature (°C)	−10
	Density (kg/m^3^)	1010
	Dielectric properties	47.1 − i × 21.4 [36] (where i = $\sqrt{-1}$)
	Specific heat capacity	Temperature-dependent [36]
	Thermal conductivity	Temperature-dependent [36]
Operating conditions	Total heating time (min)	6
	Microwave frequency (GHz)	2.45
	Microwave power (W)	1200

**Table 2:** Summary of the optimized thickness using the integrated approach and corresponding HUI change compared with the parametric sweep approach.

Sample Shape	Training Data Type	Replicate	Optimal Thickness, cm *	Optimized Thickness, cm **	HUI Change, % ***
Rectangular	Evenly distributed	Rep 1	1.60	1.55	−2.5
		Rep 2		1.56	−3.2
		Rep 3		1.53	−2.6
	Randomly selected	Rep 1	1.60	1.49	+2.7
		Rep 2		1.54	−3.5
		Rep 3		1.53	−1.7
Conical	Evenly distributed	Rep 1	1.70	1.74	−3.7
		Rep 2		1.71	−2.5
		Rep 3		1.72	−0.8
Elliptical	Evenly distributed	Rep 1	1.50	1.47	−1.9
		Rep 2		1.41	+4.3
		Rep 3		1.54	−2.6

* determined from the parametric sweep approach, used as a reference value; ** determined from the integrated approach; *** “−” means that the HUI value is smaller than the optimal result, indicating an improvement.

**Table 3:** Optimization efficiency.

Assessment of Optimization Efficiency	Sample Shape and Training Data Type			
	Rectangular		Conical	Elliptical
	Evenly Distributed	Randomly Selected	Evenly Distributed	Evenly Distributed
Average number of models in one optimization process *	15.7 (±2.3)	12.3 (±1.2)	11.0 (±0.0)	12.3 (±1.2)
Improvement of optimization efficiency **	45.9%	57.6%	62.1%	57.6%

* Including training models, value expressed as mean ± standard deviation for three replications; ** The number of parametric sweep models (29) was used as a reference.

## Data Availability

The data presented in this study are available on request from the corresponding authors.
